# Promoting Alcohol Reduction in Non-Treatment Seeking parents (PAReNTS): a protocol for a pilot feasibility cluster randomised controlled trial of alcohol screening and brief interventions to reduce parental alcohol use disorders in vulnerable families

**DOI:** 10.1186/s40814-018-0305-5

**Published:** 2018-06-09

**Authors:** Ruth McGovern, Elaine Stamp, Mehdi Javanbakht, Elaine McColl, Matthew Hickman, Eileen Kaner

**Affiliations:** 1 Institute of Health & Society, Newcastle University, Newcastle upon Tyne, UK; 2 School of Social and Community Medicine, University of Bristol, Bristol, UK

**Keywords:** Alcohol, Brief interventions, Parent, Vulnerable families, Children’s social care

## Abstract

**Background:**

Research estimates that 30% of children under the age of 16 years in the UK live with at least one parent with an alcohol use disorder (AUD). Parental AUDs are associated with adverse childhood experiences and poorer outcomes for children. The PAReNTS (Promoting Alcohol Reduction in Non-Treatment Seeking parents) trial aims to examine the feasibility and acceptability of a randomised controlled trial of brief alcohol interventions to reduce parental alcohol misuse.

**Methods:**

The cluster randomised controlled trial will be conducted within early help family support and children’s social care services in three local authorities in the North East of England: Newcastle, Durham and North Tyneside. All eligible parents the caseloads of participating practitioners will be screened for an AUD using the Alcohol Use Disorder Identification Test – Consumption (AUDIT-C) screening tool by the social care practitioners within routine appointments. All parents who score 5 or more on the AUDIT-C will be invited to participate in the trial. Consenting participants will complete a baseline questionnaire before receiving one of three randomised interventions: (i) healthy lifestyle leaflet (control intervention); (ii) a brief alcohol advice intervention delivered by the social care practitioner plus healthy lifestyle leaflet; (iii) a brief alcohol advice intervention delivered by the social care practitioner, healthy lifestyle leaflet plus a 40-min behaviour change intervention with an optional review session delivered by the local alcohol service. Follow-up data will be collected 6 and 12 months post recruitment. A linked qualitative study will explore participating parent and practitioner views on the acceptability of trial processes and interventions.

**Discussion:**

The PAReNTS trial will provide a robust estimate of recruitment, retention and consent rates in order to inform the design of a future definitive study examining the effectiveness and cost-effectiveness of alcohol screening and brief interventions to reduce parental AUDs within vulnerable families.

**Trial registration:**

ISRCTN registry ISRCTN60291091; protocol version 2; 17.10.2016

**Electronic supplementary material:**

The online version of this article (10.1186/s40814-018-0305-5) contains supplementary material, which is available to authorized users.

## Background

Approximately 30% (3.3–3.5 million) of children under 16 years of age [1] and 12% (93,517) of infants under 1 year of age [2] in the UK live with a parent who has an alcohol use disorder (AUD). There are three categories of AUD: hazardous, harmful and dependent drinking. Hazardous drinking is a repeated pattern of drinking that increases the risk of physical or psychological problems whilst harmful drinking is defined by the presence of these problems [3]. Alcohol dependence is defined as a cluster of physiological, behavioural and cognitive phenomena in which the use of alcohol takes on much higher priority for a given individual than other behaviours that once had greater value [3]. There is well-established evidence documenting the harmful effect that parental alcohol dependency has upon the child throughout their life course [4]. Children whose parents are dependent upon alcohol are more likely to suffer an injury [5], as well as an injury of greater severity [6], and to experience health problems to which their parents may not respond effectively [4]. Pre-school children are at risk of delays in cognitive and language development [7] and a greater likelihood of education deficits [8]. They can go on to have lower educational performance in adolescence [9], resulting in poor life chances [4]. However, the association between parental alcohol misuse and adverse outcomes in children is not restricted to dependent levels of use [10]. Parental AUD below dependent levels is also associated with childhood accidental injury [11, 12], hospital admissions [13], externalising difficulties [14–16] and lower school performance [17]. Children whose parents display non-dependent AUD are themselves likely to go on to early onset alcohol use [18], regular alcohol consumption [19] and alcohol intoxication [20].

Due to the potential negative impact on the child, parental AUDs are emphasised as a parental risk factor within guidance on safeguarding children [21]. In England, parental alcohol misuse was identified as a risk factor in 19% of child in need assessments [22]. A study of cases allocated for a long-term social work intervention found that parental ‘alcohol misuse’ (non-defined) was a concern in 68 out of the 290 cases (23%) [23]. Fifty-six percent of mothers who have been involved in recurrent care proceedings were engaged in misuse of alcohol and others drugs during the index proceedings [24]. Moreover, parental alcohol misuse was recorded in 37% of serious case reviews (local enquiry following the death or serious harm to a child where abuse or neglect are known or suspected) [25]. Whilst practitioners within children’s social care consider it a legitimate part of their role to ask parents about their alcohol use, they experience difficulty in identifying parents who have AUDs [26, 27]. Practitioners typically rely upon observations of parental intoxication or informal assessment of alcohol problems. Such methods are unlikely to identify parental AUDs below the diagnostic threshold for dependence, resulting in a dichotomous assessment of the presence or absence of alcohol problems, rather than recognition of a continuum of risk. Indeed, the lack of valid alcohol and other drug use assessment tools within social care settings has been highlighted in research [26]. Furthermore, those parents that are identified as misusing alcohol often do not receive an intervention, with parents expressing reluctance to engage with specialist drug and alcohol treatment providers as they did not perceive themselves as having a substance misuse problem [23]. Given the propensity of harms to children associated with parental alcohol misuse, as well as the harms to the individual drinker, it is both a public health [28] and safeguarding priority [29] to address parental AUDs. In a major review of UK child protection services, preventive rather than reactive services were highlighted to be more effective in reducing abuse and neglect of children [30]. The importance of intervening early in parental AUDs amongst other parental stressors has been highlighted in guidance for health, social care and third sector partners [21, 30, 31], yet recent inspections have found that services do not sufficiently recognise the needs of families affected by parental alcohol misuse [32, 33].

A number of trials have examined the effectiveness of psychological interventions in reducing parental alcohol and drug misuse. Whilst the findings of these trials are mixed, there is some evidence that interventions with a focus upon the family or those with clear extrinsic motivation for the parent (such as those linked to care proceedings) are effective at reducing parental alcohol and drug misuse. Statistically significant reductions in parental substance use were found in interventions which included parental skill training and education [34–37], those that were ecological or systemic in their approach [38, 39] and those that involved relational therapy [36, 40]. However, much of this research was reactive, focused upon parents who were dependent upon alcohol or other drugs and whose use of substances were directly related to incidents of child abuse and neglect.

There is a paucity of research on preventive interventions to reduce AUD in parents [41]. There is, however, a large amount of high-quality evidence to support the effectiveness of alcohol screening and brief interventions with adults who have AUDs [42]. Indeed, the evidence base for brief interventions represents the largest, most robust body of evidence for alcohol interventions [43]. Most of this evidence base has been developed in primary care, however; other settings have learned from these studies and examined the benefits to their patients. Indeed, there have been a number of systematic reviews and individual studies of brief interventions in emergency departments [44, 45] and with other populations such as young people [46, 47] and pregnant women [48, 49] showing some beneficial effect. However, there are no studies examining the effectiveness of alcohol screening and brief interventions within social care settings or brief interventions delivered to parent populations.

## Aim

The PAReNTS (Promoting Alcohol Reduction in Non-Treatment Seeking parents) study is a pilot feasibility randomised controlled trial of brief alcohol interventions to reduce parental AUD. The trial aims to investigate whether it is possible to recruit parents involved with early help family support (voluntary support) or children’s social care (statutory intervention) who have an AUD, and whether these parents can be retained at the 12-month follow-up. The feasibility and acceptability of the study interventions and trial procedures will be examined. If feasibility and acceptability is shown, the pilot feasibility trial will inform the protocol for a definitive cluster randomised controlled trial.

The specific objectives of this pilot feasibility trial are:

- To assess local authority engagement in an alcohol trial that uses a randomised controlled design
- To estimate rates of participant eligibility, recruitment, randomisation, retention and response to outcome measures within the baseline and follow-up questionnaires to inform sample size calculations for a future definitive trial
- To assess engagement and participation with the alcohol interventions
- To develop cost assessment tools, assess intervention delivery costs and carry out a value of information analysis to inform a definitive trial
- To apply pre-specified ‘stop/go’ criteria to determine if a definitive multi-centre randomised controlled trial is feasible and, if so, to develop a full trial protocol.

A clear success criteria (‘go’) would be meeting of all of the targets of recruitment of three local authorities as research sites from within the North East region of England; ≥ 60% of eligible participants consenting to pilot feasibility trial; ≥ 60% of consenting participants accepting/attending intervention sessions; retention of ≥ 60% of consented participants for provision of key outcome data at 12 months. These are common parameters considered when decided upon progression to definitive trial [50]. Whilst within randomised controlled trials of brief alcohol interventions in primary care settings often report a consent rate of 80% and aim to retain 70% of participants at the 12-month follow-up [51], parents involved in early help family support and children’s social care are a vulnerable population and external factors within their daily living may reduce the likelihood of providing consent to participate in the trial and of being retained at follow-up. As such, more cautious success criteria have been suggested. Receipt of interventions delivered opportunistically is typically high in trials of brief interventions (99%); however, attendance at scheduled brief interventions wherein a participant is required to return at a later date for an appointment is much lower (around 50–60%) [51–53]. To allow for this variation between interventions, the intervention will be considered to have met the success criteria if ≥ 60% of randomised participants in each arm receive the intervention. Where marginal results are reached (recruitment of two local authorities; 59–40% of eligible participants consenting to pilot feasibility trial; retention of 59–50% of consented participants at 12 months; 59–30% of participants receiving a randomised intervention), a process for decision making after pilot and feasibility trials (ADePT) [54] will be followed, systematically appraising the problems and potential solutions within the linked process evaluation.

## Methods

### Recruitment and screening

The trial will be conducted within early help family support and children’s social care services in three local authorities in the North East of England: Newcastle, Durham and North Tyneside. These particular local authorities were selected due to their geographical and service model variability. Participating practitioners within early help family support and children’s social care services will screen all eligible parents on their caseloads for AUDs within routine appointments. To be eligible, parents must be 18 years or over and be able to give informed consent in English. A broad definition of ‘parent’ will be applied wherein any adult who fulfils a parenting role (as identified by the additional early help family support and/or children’s social care assessment) for a child aged 0–17 years will be considered eligible for the study. This includes both mothers and fathers, step parents and other caregivers. Parents who are attending drug and alcohol services; who have severe, chronic or acute mental health problems or are severely distressed; or whose child is placed on an emergency protection order will be ineligible to participate in the study. Social care practitioners will record demographics of all eligible parents and provide details of the reason for social care involvement. The social care practitioner will administer the Alcohol Use Disorder Identification Test – Consumption (AUDIT-C) screening tool, which measures alcohol consumption [55]. The AUDIT-C is a three-item tool that has been found to have comparable sensitivity to the 10-item AUDIT in detecting heavy drinking; it performs better than the 10-item AUDIT in detecting alcohol dependence in women and has greater efficiency in screening hazardous alcohol use in men [56]. The tool was considered to be preferable to the 10-item version for use in early help family support and children’s social care setting. Parents within this setting are likely to be concerned that their parenting capacity will be negatively judged and may therefore be reluctant to provide an accurate response to the dependence and control items of the 10-item AUDIT. Moreover, the use of the AUDIT-C is in-line with usual practice in social care. It has been validated using a cut-off score of ≥ 5 in European general populations [57], men and middle-aged women [58]. Those parents screening positive for AUD on the AUDIT-C (an AUDIT score of ≥ 5) [59] will be invited to participate in the trial by the social care practitioner. The social care practitioner will provide a participant information leaflet to the parent, explain the trial and seek informed written consent for participation. In families where multiple caregivers screen positive and agree to participate, the parents will be recruited as individual cases.

### Randomisation and blinding

The study will employ a cluster randomisation design, with the practitioners being the unit of randomisation. As a single practitioner works with all family members, this will prevent within-family contamination (i.e. if multiple members of one family are recruited to the trial, all family members will receive the same intervention), as well as reducing contamination caused at a practitioner level. The intervention delivered by the early help family support and social care practitioners is provided to the participant immediately following recruited and within the same contact. As such, randomisation will not be compromised by a change in the participant’s allocated practitioner. The practitioners will be randomised to one of the three trial arms in a 1:1 ratio using block randomisation. The study statistician will allocate practitioners to trial arms using a randomised sequence created in Stata 14 [60]. The randomisation will be stratified by a local authority area and practitioner role (early help practitioner, family support worker, social worker within the assisted and support year in employment and progressed social worker), with equal probabilities for each of the three arms and randomly generated varying block size. Service level (early help family support or statutory children’s social care) should be balanced across the strata by the randomisation. Blinding of group allocation will not be possible for the participants or the researcher completing follow-up questionnaires. The study statistician and health economist will be blind to group allocation throughout analysis; participants and practitioners will only be identified by study numbers. Within a definitive trial, researchers, statisticians and health economists will be blinded.

### Sample size

**Fig. 1:**
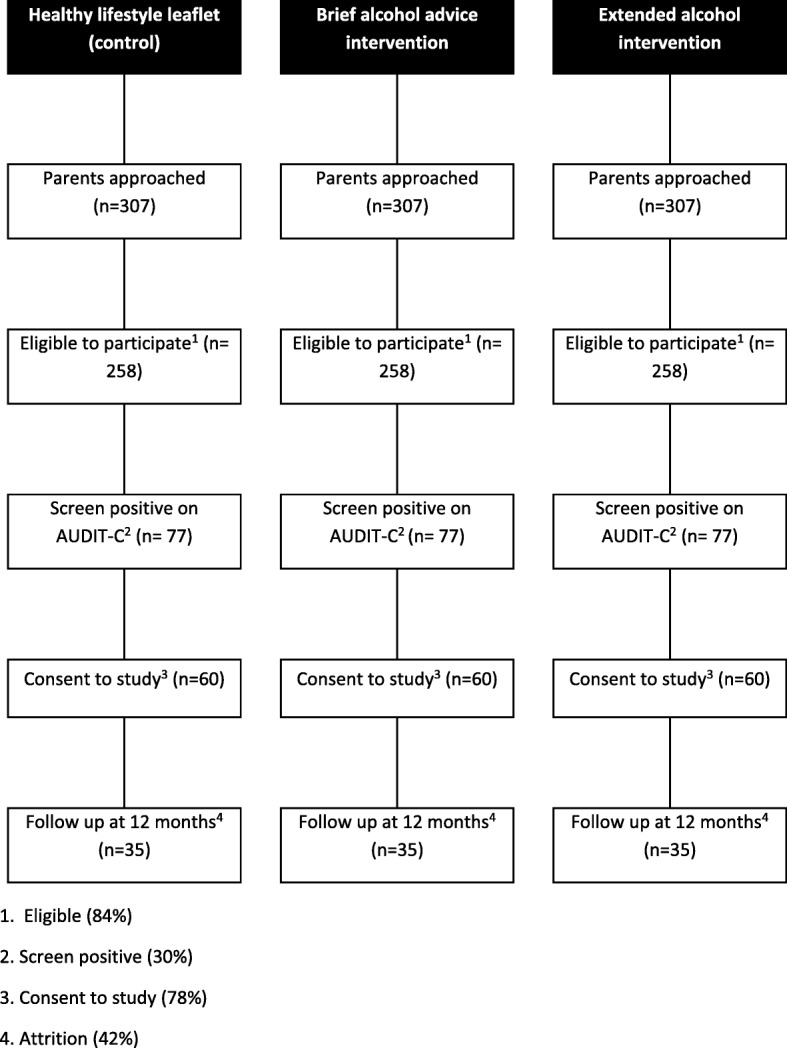
Recruitment flowchart

As this is a pilot feasibility trial and not an outcome evaluation, no formal power calculation is required [61]. Rather, the function of a pilot feasibility trial is to provide data that can inform a power calculation for a future definitive trial. For the pilot trial, a minimum of 35 respondents in each trial arm at the 12-month follow-up is required to estimate the critical parameters to the necessary degree of precision for a continuous primary outcome (number of occasions drinking 5+ standard drink units in a single occasion as derived from the TLFB/30) [62]. Attrition of 33% at the 12-month follow-up is generally assumed within feasibility trials [63]. However, due to a paucity of trial-based research conducted with parents involved in early help family support and children’s social care, a cautious 42% loss to follow-up has been applied. Consequently, the sample size to be recruited will be inflated to 60 parents in each of the three arms (Please see Fig. 1).

To achieve this sample, we estimate that social care practitioners within three local authorities will need to approach 921 parents. We expect 84% of parents will meet the inclusion criteria for the trial, similar to rates of eligibility within primary care populations [51]. Based upon Manning et al. [1], we estimate that 30% of parents will score 5 or more on the AUDIT-C and, similar to a brief alcohol intervention trial with pregnant women [49], 78% will provide informed consent to participate in the trial. We estimate that we will need to recruit 45 social care practitioners across the three local authority areas (15 practitioner per local authority, with five practitioners per local authority randomised per trial arm), each approaching 21 parents and recruiting four parents to the trial.

### Consent

Social care practitioners will provide all parents on their caseloads with an information leaflet about the study a minimum of 24 h in advance of conducting alcohol screening. After administering the AUDIT-C, social care practitioners will explain the study to eligible parents who screen positive for an AUD and answer any questions they may have. If the parent decides to participate, they will be asked to provide written consent, which will be recorded on a consent form (please see consent form in the Additional file 1: Appendix). Additional consent will also be sought for qualitative interviews which will be conducted within the process evaluation, and audio-recording of intervention sessions (to enable fidelity assessments). The consent form will include a unique identifier which enables the consent to be linked to the anonymised personal data gathered within the baseline questionnaire.

### Baseline and follow-up procedure

After informed consent has been obtained, but before intervention delivery, the social care practitioner will collect baseline information from the parent. All participants who completed baseline questionnaires will be contacted by telephone, letter or email by a member of the research team, at 6 and 12 months post recruitment, and invited to complete a follow-up questionnaire. A response window of 8 weeks will be in place to maximise follow-up. Participants who do not complete the 6-month follow-up will still be invited to complete the 12-month follow-up. The questionnaire will be administered in person or over the phone by the researcher or self-completed via post, email or the web (depending upon participant preference). If after a minimum of three repeated attempts the researcher is not able to contact the participant by their preferred method, alternative methods will be attempted (telephone, postal or email contact). Where all attempts to contact the participant fail, effort will be made to make contact with the participant via the recruiting social care practitioner.

### Measures

The following measures will be administered at baseline and at both follow-ups:

- Remaining 7 questions of the *AUDIT*, 10-item tool, will be used to measure alcohol problems. The AUDIT is the *gold standard* alcohol screening tool [64]. In addition, participants will be asked to state their average weekly spend on alcohol.
- Family functioning will be assessed with the *family perceptions scale* (FPS). The FPS is a 29-item tool across five domains (nurture, problem solving, expressed emotion, behavioural boundaries and responsibility) [65]
- Mental health and well-being will be assessed using the *Warwick Edinburgh Mental Well-Being Scale* (WEMWBS). The WEMWBS is a 14-item scale of mental well-being covering subjective well-being and psychological functioning, in which all items are worded positively and address aspects of positive mental health. The tool has been used extensively with adults [66]
- Health-related quality of life (HRQoL) will be assessed using the *European Quality of Life—5 Dimensions—5 Levels* (EQ-5D-5L) instrument. EQ-5D has been developed by the EuroQol Group to provide a simple, generic measure of health for clinical and economic appraisal, where health is characterised on five dimensions (mobility, self-care, ability to undertake usual activities, pain, anxiety/depression) [67].

The following measures will be administered at the 6- and 12-month follow-up:

- *Alcohol problems questionnaire*—this is a clinical instrument for measuring alcohol-related problems. It has been used to study the relationship between alcohol-related problems and dependence within the bi-axial model [68]
- A modified *Client Service Receipt Inventory—*this is a tool used to comprehensive assess service usage to enable economic analysis [69]
- Local authority system-held information relating to case allocation, legal and care status of the child will be collected from children’s social care at the 6- and 12-month follow-up.

The following measure will be administered at the 12-month follow-up only:

- *30-day timeline follow back* (TLFB30). The TLFB30 is an established valid and reliable method of ascertaining alcohol consumption in adult populations over a reference period ranging from 7 to 365 days [70].

Please see Table 1 for further details.

**Table 1:** Schedule of events

Study period						
Research phase	Recruitment	Baseline	Session 1	Session 2	6-month follow-up	12-month follow-up
Informed consent	X					
Demographics		X				
Eligibility assessment	X	X				
AUDIT (AUDIT-C as screening tool; remaining questions of 10-item tool administered at baseline)	X					
Control group Healthy lifestyle leaflet	X					
Intervention 1 Brief alcohol advice plus healthy lifestyle leaflet	X		X			
Intervention 2 Extended alcohol intervention plus brief alcohol advice plus healthy lifestyle leaflet	X		X	X		
AUDIT (10-item)		X			X	X
Estimated spend per week on alcohol		X			X	X
WEMWBS		X			X	X
EQ-5D-5L		X			X	X
FAD		X			X	X
TLFB-30						X
APQ					X	X
Use of health and social services					X	X
Case allocation, legal and care status					X	X
Qualitative interviews with participating practitioners					X	X^a^
Qualitative						

^a^Qualitative interview participants will be interviewed on one occasion only at varying stages of the follow-up

All identifiable data collected will be separated from personal data to maintain confidentiality and a unique reference number allocated to enable data linkage across sources and time periods. All data will be stored on a secure server, with identifiable data being password protected and stored.

### Interventions

#### Control

Participants within the control group will receive a healthy lifestyle leaflet produced by Public Health England. This leaflet provides information on small lifestyle changes that can improve health and reduce illness. This includes brief information that drinking less alcohol can boost energy and improve sleep quality as well as information about diet, exercise, sleep, stress and health.

#### Brief alcohol advice

Parents within the brief alcohol advice group will be screened and receive personalised feedback and a 20-min brief alcohol advice intervention from their social care practitioner immediately following completion of the baseline questionnaire. The intervention will be based upon the ‘How much is too much?’ brief intervention programme (level 1), an evidence-based programme highlighted by the National Institute for Heath and Clinical Excellence alcohol prevention guidance (PH24) [71]; it has been modified for delivery with parents involved in early help family support and children’s social care services. Advice is provided on recommended levels of alcohol use. The intervention takes a strengths-based approach and therefore starts by considering family-level protective factors which may mitigate the impact of alcohol misuse upon the family. Tailored advice on the risks of drinking above the recommended levels for the parent, the child and the household as well as the impact upon family activities is provided. The parent is then encouraged to complete a ‘parent plan’. This plan involves the parent setting goals to either reduce alcohol use or to minimise the impact of their alcohol use upon themselves and their child (Additional file 2). Should participants become distressed or express a desire to withdraw, the intervention will be discontinued .

#### Extended alcohol intervention

Participants within the extended alcohol intervention will be screened and receive personalised feedback and a 20-min brief advice intervention as detailed above. In addition, the participants will be invited to attend the local alcohol treatment service within 2 weeks to receive the extended intervention. Participants can choose to decline attendance at the alcohol treatment provider. The extended alcohol intervention consists of one 40-min session with a further optional review session. The approach involves a patient-centred counselling technique, informed by the principles of motivational interviewing [42]. This approach is based upon the ‘How much is too much?’ brief intervention programme (level 2), adapted for delivery with parents involved in early help family support and children’s social care services. It aims to introduce and evoke change by giving the parent the opportunity to explore their alcohol use as well as their motivations and strategies for change. The parent is asked to explore a typical day in their family, a typical day when they consume alcohol and a typical day after they have consumed alcohol. Motivational domains are developed through exploration of importance and confidence to change as well as the pros and cons of change for the parent and the child. Once motivation to change is developed, the parent is then encouraged to make a practical and personalised plan to reduce their alcohol consumption. A further optional review session may be arranged should the participant wish to return to the service to review the progress they have made against their personalised plan (Additional file 3). Should participants become distressed or express a desire to withdraw, the intervention will be discontinued.

### Training and fidelity

The unit of randomisation within this cluster randomised controlled trial will be the social care practitioner. Practitioners will only be trained in the intervention to which they are randomised, with practitioners in the control group receiving no intervention training. This approach will restrict their knowledge of the skills and techniques used in other intervention groups and is therefore expected to reduce the likelihood of contamination between groups. Within each local authority area, a specialist alcohol treatment provider will be recruited to deliver the extended alcohol intervention. All social care practitioners will be trained in general trial procedures and their own randomised intervention. This training for the control group will last 1 h and will focus on the aims of the trial, trial procedures and issuing the healthy lifestyle leaflet. The training for the social care practitioners within both the brief alcohol advice and extended alcohol intervention groups will last around 4 h and will cover the impact of parental alcohol misuse upon children, the aims of the trial, trial procedures and delivery of the brief alcohol advice intervention. Practitioners within the extended alcohol intervention group will also receive information on the process for referring participants to the alcohol treatment service and the content of the extended alcohol intervention. Specialist alcohol practitioners will be trained to deliver the extended alcohol intervention. This training will last around 2 h and will cover the aims of the trial and delivery of the extended intervention.

Twenty percent of the brief and extended alcohol interventions (*n* = 12 of each intervention) will be audio-recorded and assessed for intervention fidelity. We will apply an a priori designed checklist tool to assess the fidelity of intervention delivery of the brief intervention sessions. We will assess the extended intervention using a validated rating scale (BECCI) [72].

### Statistical analyses

As a pilot feasibility trial, no formal hypothesis is to be tested. Rather, the aim of the trial is to provide robust estimate of rates of recruitment, retention and consent to inform the design of a future definitive study. Descriptive analysis will include participant characteristics (age, sex, educational attainment, number of children and whether the parent is involved in early help family support or statutory children’s services), the number and percentages recruited and retained at both follow-up points and variability in study measures. If a definitive trial is judged to be feasible, a decision will be made on a primary outcome measure. The sample size calculation within the definitive trial would follow the principle described within the extension of the CONSORT 2010 statement [73].

### Economic evaluation

The purpose of a definitive economic evaluation is to assess benefits as well as costs to patients and their families/carers, the NHS and personal social services (societal perspective). Health economic assessment will consider real costs (training of practitioners, screening of parents, intervention delivery) and outcomes (reduced alcohol consumption, reduced problems, service usage). Data on health consequences will be collected by means of participant completed questionnaires collected at baseline and the 12-month follow-up to capture changes in health-related quality of life outcomes (measured by EQ-5D-5L). Responses to the EQ-5D-5L will be used to calculate quality adjusted life years (QALYs). The CSRI will be modified for the definitive trial in response to data collected within the current study, to make it relevant to the study population. The associated costs will relate to resources required to provide the intervention and usage of NHS, public and personal social services and patient cost during the follow-up period. We will ascertain data completeness of the instruments and any potential bias in the completion of follow-up data to inform the choice of instruments in a future trial. The majority of the outcome data will be presented in simple descriptive tables presenting percentages, means and standard deviations or five-number summary (as appropriate), for each arm of the study. The findings from the pilot feasibility trial will then be incorporated into a probabilistic mathematical decision analytic model to provide preliminary estimates of cost, effectiveness and relative cost-effectiveness and assess the value of information (VoI) for a definitive study. The VOI will be used to inform the design, choice of primary outcome, necessary sample size and approach to the analysis, of the future definitive trial. The VOI will also be used to estimate the expected value of sampling information (EVSI). The EVSI will quantify the value of reducing uncertainty via collection of additional data in a definitive trial.

### Realist process evaluation

We will conduct a process evaluation informed by realist principles. This will enable the theorising and empirical examination of the underlying mechanisms of the brief intervention [74] and uncover context-mechanism-outcome configurations [74, 75], which seeks to understand what works within an intervention, for whom, and under what circumstance [76]. Individual, in-depth interviews will be conducted with a purposive sample of participating parents and practitioners. Interviews will continue until data saturation is reached; it is expected that approximately 20–25 participating parents and 15–20 practitioners will be required. We will aim for a maximum variation sample, to achieve a broad perspective on these issues. Parents will be sampled based upon their parental role, stage and reason for social care involvement, study arm, AUDIT-C score, and LA of residence. Social care practitioners will be sampled on study arm, employing LA, early help family support and statutory children’s social care, gender, and number of parents screened and recruited into the trial. Alcohol practitioners who have experience of delivering the extended alcohol intervention will be sampled based on geographical area and gender. A semi-structured interview approach will be adopted, enabling the interviewer to gather the information necessary to respond to the aims of the feasibility study, whilst also allowing new themes to emerge. Interviews with practitioners will examine the feasibility and acceptability of implementation; the structures, resources, and process through which delivery was achieved will be considered alongside contextual and individual barriers and facilitators to delivering the intervention. Intervention adaptations will be examined with consideration of whether any identified variation from intended delivery represents innovation or intervention drift. Further, interviews will examine potential delivery systems and structures for future implementation, as well as perceived restrictions linked to the implementation and commissioning of interventions with this population. The interviews with participants will explore previous involvement with early help family support and/or children’s social care, experience of trial participation as well as the intervention, engagement with the intervention, and the perceived impacts thereof (both intended and unintended) in order to develop understanding of the mechanisms of change. Contextual factors will be examined from both the participant and practitioner viewpoint, exploring issues relating to consent, the potential for coercion around participation, disclosure of alcohol consumption within the context of early help family support and children’s social care and contextual factors which shape the theory of how the intervention works. Interviews will be audio-recorded and transcribed verbatim. Data will be subject to framework analysis, which is appropriate for qualitative health research with objectives linked to quantitative investigation [77]. This analytic strategy is characterised by a more deductive than inductive approach, whereby analysis is structured around given themes so that findings have detailed relevance to applied research questions [78, 79]. All transcripts will be repeatedly read and coded using a framework of a priori headings. The findings will inform the refinement of the intervention logic and dark logic models [80], wherein the intended (logic model) and unintended adverse impacts (dark logic model) of the intervention will be anticipated and monitored. In doing so, the process evaluation will examine what works for who under what circumstance [74]. The findings of the realistic process evaluation will augment the findings of the feasibility pilot trial and inform the design of the definitive trial.

### Timescale

Trial recruitment commenced Oct 2017 and is currently ongoing. The duration of the trial is 24 months.

### Trial registration

The trial is registered with the ISRCTN registry. The trial reference is ISRCTN60291091.

## Discussion

It is important to perform a pilot feasibility trial when the logistics of a large-scale trial are unclear [62, 63]. Whilst there is a large evidence of effectiveness of brief intervention within primary care [81] and other health settings [44], this is the first trial of alcohol brief interventions with parents in a social care setting. Trials of other psychosocial interventions with substance misusing parents have mostly included parents who are dependent upon substances rather than being hazardous or harmful alcohol users. Typically, the participants of these trials are mothers, with a lack of trials including fathers. Further, these trials have mostly been conducted within the USA, with important social care and child welfare system differences.

The UK early help family support and children’s social care setting is different to healthcare settings. Parents may be unwilling to disclose their alcohol use, particularly within statutory social care settings where fear of judgement may be high. Randomised controlled trials are uncommon in the social care setting; busy practitioners may find it difficult to implement trial processes within their routine practice or may not be motivated to do so. Parents may be unwilling to consent to participate in a trial of brief alcohol interventions, follow-up rates are unknown in this population and there may be variation in recruitment and retention rates between mothers and fathers. Little is understood about the most appropriate professional (early help family support practitioner, social worker, family support worker) to deliver an alcohol intervention to parents. This pilot feasibility trial will indicate whether a definitive trial of the effectiveness and cost-effectiveness of alcohol screening and brief intervention with parents within early help family support and children’s social care setting is feasible and, if so, how such a trial should be designed.

A key consideration in the design of a potential future trial is whether the trial should consist of two or three arms. The decision to include three arms within the current pilot feasibility trial is based upon the sensitivity of the topic of alcohol misuse by parents within this setting. The social care practitioner has an ongoing relationship with the parent; this relationship may help or hinder the delivery of an alcohol intervention. The social care practitioner has a responsibility to respond to a wide range of family needs and the opportunistic delivery of a brief alcohol advice intervention may be experienced as an appropriate activity. Conversely, the context of the relationship might create a threat that is not conducive of behaviour change. A specialist alcohol practitioner will be skilful in behaviour change techniques and may offer a relationship wherein the parent feels more able to disclose alcohol misuse. Yet hazardous and harmful alcohol users may not perceive their alcohol use as warranting attendance at specialist alcohol services. Intervention piloting will also enable intervention detail to be refined. These issues will be examined in-depth within the realist evaluation, which will add rich understanding to the quantitative data gathered in the pilot feasibility trial.

If recruitment and retention success criteria are also reached, we plan to proceed to a RCT of the effectiveness and cost-effectiveness of brief alcohol interventions to reduce parental AUD. The mixed methods findings of this study will inform the development of a protocol for the definitive trial. This will include a sample size calculation, refined logic and dark logic models. The current study should also uncover context-mechanism-outcome-configurations so that we can theorise what will work for whom, and under what circumstance. As such, this study will usefully extend the evidence base in this field at an international level. Authorship of future published outputs will include authors who have made a substantial contribution to the conception or design of the work; or the acquisition, analysis or interpretation of data for the work, including drafting and authorising of manuscripts. Trial findings will also be communicated to trial participants within a lay summary.

## Additional files

Additional file 1: Participant consent sheet. (DOCX 1900 kb)

Additional file 2: Brief alcohol advice intervention. (DOCX 1500 kb)

Additional file 3: Extended alcohol intervention. (DOCX 3600 kb)

## Abbreviations

ADePT: A process for decision making after pilot and feasibility trials
AUDIT: Alcohol Use Disorder Identification Test
AUDIT-C: Alcohol Use Disorder Identification Test – Consumption
AUDs: Alcohol use disorders
BECCI: Behaviour change counselling index
EQ-5D-5L: European Quality of Life—5 Dimensions—5 Levels
EVSI: Expected value of sampling information
FPS: Family perceptions scale
HRQoL: Health-related quality of life
LA: Local authority
PAReNTS: Promoting Alcohol Reduction in Non-Treatment Seeking parents
QALYs: Quality adjusted life years
TLFB30: 30-Day timeline follow back
VoI: Value of information
WEMWBS: Warwick Edinburgh Mental Well-Being Scale
