# Supplementary material for: Promoting Alcohol Reduction in Non-Treatment Seeking parents (PAReNTS): a protocol for a pilot feasibility cluster randomised controlled trial of alcohol screening and brief interventions to reduce parental alcohol use disorders in vulnerable families
Source: Pilot Feasibility Stud. 2018 Jun 9;4:111. doi: 10.1186/s40814-018-0305-5 (PMC5994069; doi:10.1186/s40814-018-0305-5)
Supplement: Supplementary file 1 — Participant consent sheet. (DOCX 1900 kb) [file 40814_2018_305_MOESM1_ESM.docx]

**Participant consent form**


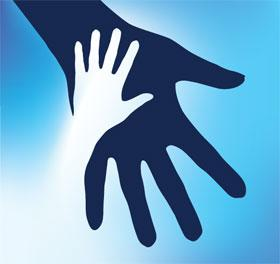

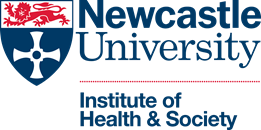

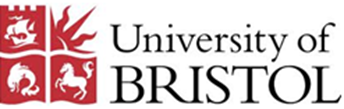
Participant ID:

| **PAReNTS**  **Promoting Alcohol Reduction in Non-Treatment Seeking Parents**  **PLEASE INITIAL EACH BOX**  **Consent Form** | |
| --- | --- |
|  |  |
| 1. I confirm that I have read the participant information leaflet dated 17.10.16 (version 2.0) for the above study. |  |
| 1. I confirm that I have had the opportunity to ask any questions about the study and any questions I have asked have been answered to my satisfaction. |  |
| 1. I understand that taking part is voluntary and that I’m free to change my mind at any time without giving any reason and without my care, services from children’s services or my legal rights being affected. |  |
| 1. I give permission for Children’s Services to share information with Newcastle University regarding the legal and care status of my child(ren) and whether my case remains active to Children’s services at 6 and 12 month follow-up points. 2. I understand that the information I give will be confidential. However, if I disclose information which causes concern about risk to myself or others (including my child/ren), I understand this information will be shared. |  |
| 1. I understand that any data created from this study will be held in a locked filing cabinet for ten years after which the data will be destroyed. All the data collected will be kept anonymous and confidential, and only members of the research team will have access to this data. 2. I understand that any data created from this study will be held in a locked filing cabinet for ten years after which the data will be destroyed. All the data collected will be kept anonymous and confidential, and only members of the research team will have access to this data. 3. ***I agree to take part in the above study. I am aware that a copy of this consent form will be provided to me for my records.***   In addition:   1. I agree to the advice and help given to me as part of this trial (by the social care practitioner and the alcohol worker if appropriate) being audio-recorded (optional). 2. I agree that a researcher can contact me at a later date to invite me to participate in an interview (optional)   Date |  |
| __________________________________  __________________________________  _____________________ |  |
| Name of Participant  Signature  Date |  |

Signature

__________________________________

_____________________

Name of Person taking consent

__________________________________
