# Supplementary figures and images for: Promoting Alcohol Reduction in Non-Treatment Seeking parents (PAReNTS): a protocol for a pilot feasibility cluster randomised controlled trial of alcohol screening and brief interventions to reduce parental alcohol use disorders in vulnerable families
Source: Pilot Feasibility Stud. 2018 Jun 9;4:111. doi: 10.1186/s40814-018-0305-5 (PMC5994069; doi:10.1186/s40814-018-0305-5)

**Brief alcohol advice**


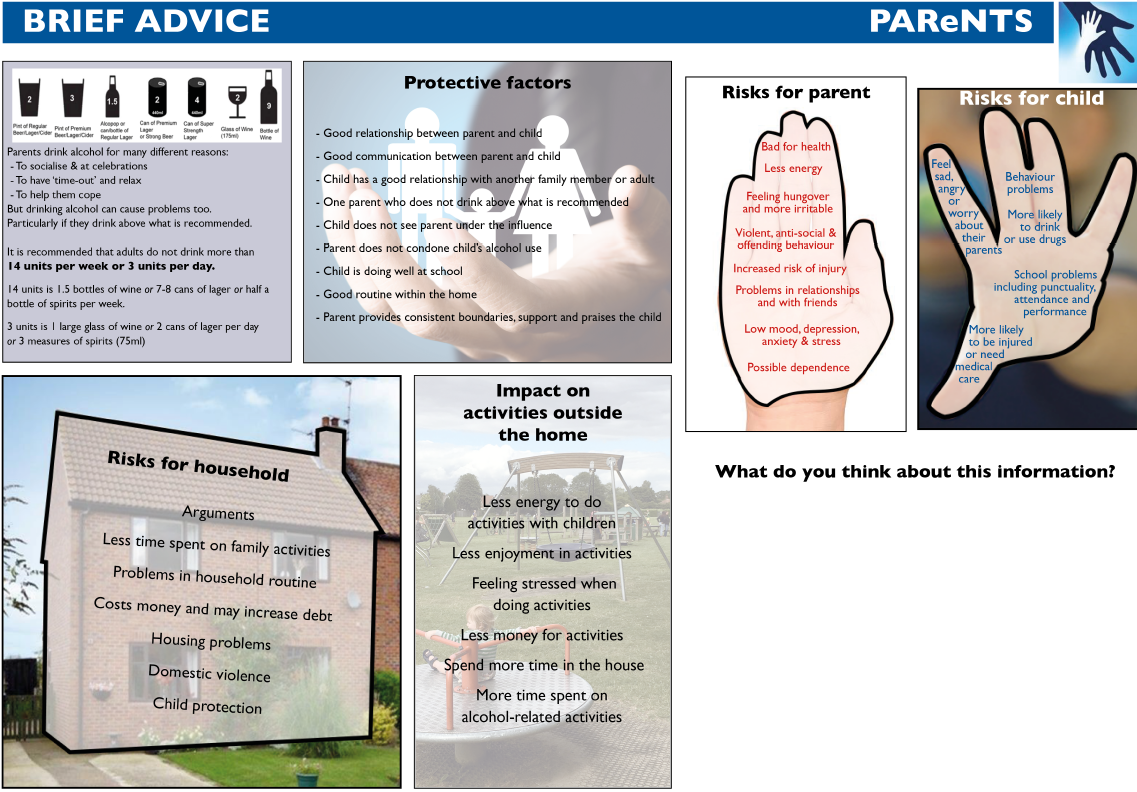


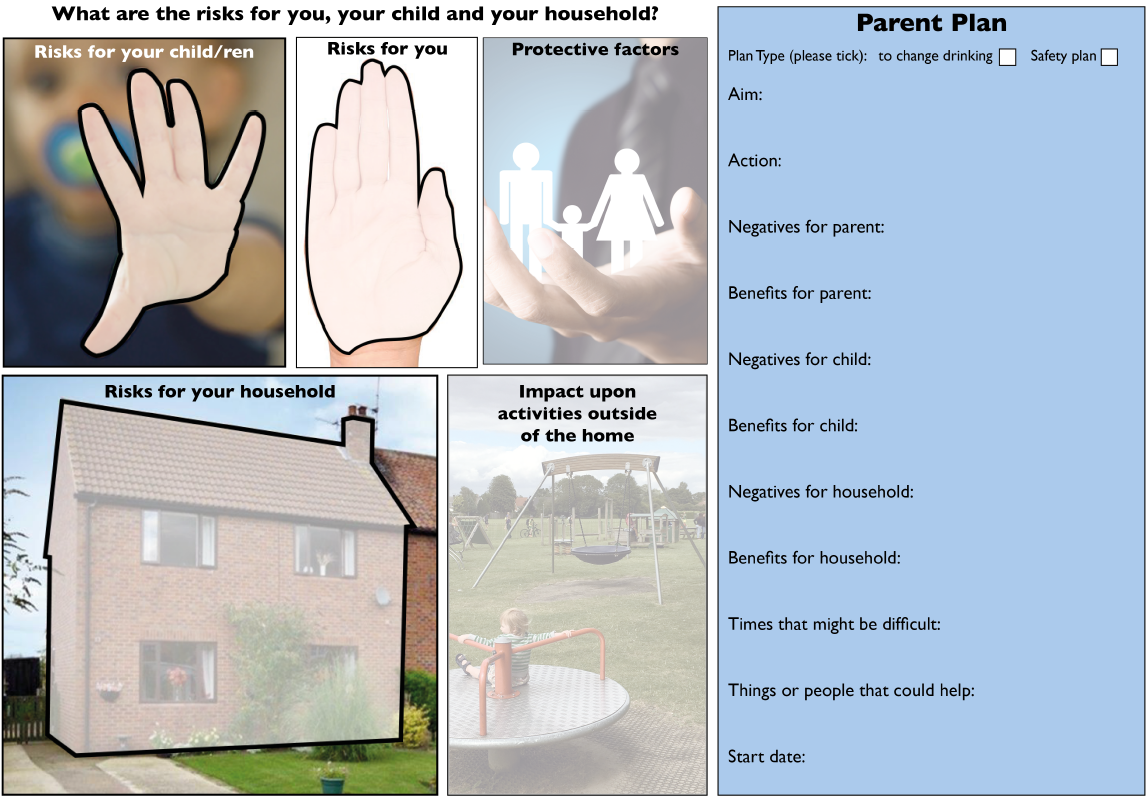

Supplement: Supplementary file 2 — Brief alcohol advice intervention. (DOCX 1500 kb) [file 40814_2018_305_MOESM2_ESM.docx]
