# Supplementary material for: Promoting Alcohol Reduction in Non-Treatment Seeking parents (PAReNTS): a protocol for a pilot feasibility cluster randomised controlled trial of alcohol screening and brief interventions to reduce parental alcohol use disorders in vulnerable families
Source: Pilot Feasibility Stud. 2018 Jun 9;4:111. doi: 10.1186/s40814-018-0305-5 (PMC5994069; doi:10.1186/s40814-018-0305-5)
Supplement: Supplementary file 3 — Extended alcohol intervention. (DOCX 3600 kb) [file 40814_2018_305_MOESM3_ESM.docx]

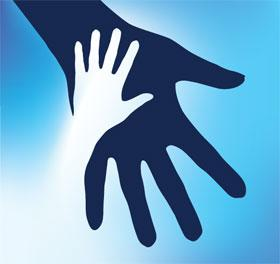
Extended alcohol intervention

**BRIEF INTERVENTION** **PAReNTS** **PAReNTS**

**Describe a typical day in your family?**

**Typical drinking day?**

What happens on a typical day when you drink?

What are the good & bad things?

**Typical day after drinking?**

What happens on day after you drink?

What are the good & the bad things?

**How important is it for you to change your drinking?**

Not important at all

Very important

**How would the people who are important to you answer this question (your child/ren, others people in your family, people outside of your family)?**

**How confident are you that you could change your drinking, if you wanted to?**

If you were to change your drinking, what would your family life be like for you?

Not so good things? Good things?

What would it be like for your child/ren and others in your family?

Not so good things? Good things?

Setting yourself a goal

3 reasons to change your drinking?

What might make you slip up?

What are the steps you could to take change your drinking?

What or who might support you?
